# Next-Generation Sequencing Reveals Field Strain Dynamics and PRRSV-2 Clearance in Gilts When Using Tylvalosin During MLV Vaccination

**DOI:** 10.3390/vaccines13101007

**Published:** 2025-09-25

**Authors:** Weixin Wu, Xiang Gao, Junfeng Gao, Zhi Lai, Xiaohong Deng, Junnan Zhang, Qiongqiong Zhou, Lei Zhou

**Affiliations:** 1 State Key Laboratory of Veterinary Public Health and Safety, College of Veterinary Medicine, China Agricultural University, Beijing 100193, China; 2 Key Laboratory of Animal Epidemiology of Ministry of Agriculture and Rural Affairs, College of Veterinary Medicine, China Agricultural University, Beijing 100193, China; 3 Research and Development Department, Shanghai Chuanghong Biotech Co., Ltd., Shanghai 201619, China; 4 Zhejiang ECO-BIOK Animal Health, Shanghai 200063, China; 5 National Engineering Laboratory for Animal Breeding and Key Laboratory of Animal Genetics, Breeding and Reproduction, Ministry of Agriculture and Rural Affairs, Department of Animal Genetics and Breeding, College of Animal Science and Technology, China Agricultural University, Beijing 100193, China

**Keywords:** porcine reproductive and respiratory syndrome virus, gilt immunization, modified live vaccine, tylvalosin, next-generation sequencing, recombination

## Abstract

Background: Porcine reproductive and respiratory syndrome virus (PRRSV) causes significant economic losses for the global swine industry. Gilt immunization using modified live virus (MLV) vaccines is crucial for herd stability, but it is complicated by frequent mixed infections of PRRSV strains on farm. This study monitored the administration of tylvalosin during a PRRSV-2 MLV (TJM) immunization program, focusing on viral dynamics and immune responses in gilts naturally exposed to co-circulating classical (GD240101) and highly pathogenic like (HP-PRRSV-like, GD240102) PRRSV strains. Methods: The animal study was approved by the Laboratory Animal Ethical Committee of China Agricultural University. One hundred gilts were randomized into control and tylvalosin groups (*n* = 50/group). All received the TJM MLV vaccination. The tylvalosin group received tylvalosin tartrate premix cyclically in-feed for three cycles. Serum and saliva samples were collected periodically. PRRSV RNA (RT-qPCR) and specific antibodies (ELISA) were assessed. Viral population dynamics (relative abundance, mutation, recombination of TJM, GD240101, and GD240102) were monitored via next-generation sequencing (NGS) on a pooled PRRSV-positive sample. Results: In this field trial where tylvalosin was used, a shorter duration of PRRSV viremia and saliva shedding was observed to compare with controls. NGS analysis showed accelerated vaccine strain (TJM) clearance in the tylvalosin group (by week 3 vs. week 9 in control). Field strain dynamics were also altered, showing a faster decline in the tylvalosin group. Antibody response uniformity was altered, with lower coefficient of variation (CV) for PRRSV and CSFV observed following tylvalosin usage. Conclusions: In gilts receiving tylvalosin for the management of bacterial pathogens during a PRRSV MLV immunization program, it was associated with accelerated viral clearance and enhanced systemic immune response uniformity under mixed-infection field conditions. NGS provides invaluable data for dissecting these complex viral dynamics. Crucially, these findings describe a biological drug–host–virus interaction and should not be interpreted as an endorsement for the prophylactic use of antimicrobials. In alignment with global antimicrobial stewardship principles, tylvalosin should be reserved for the therapeutic treatment of diagnosed bacterial diseases to mitigate the risk of promoting resistance.

## 1. Introduction

Porcine reproductive and respiratory syndrome (PRRS), caused by the PRRS virus (PRRSV), gives rise to significant economic impact on the global swine industry [1]. The mortality rate of infection with *Betaarterivirus americense* (formerly PRRSV-2) lineage 8 highly pathogenic strains (HP-PRRSV) can reach up to 100% in pigs [2,3]. The introduction of externally sourced replacement gilts is widely recognized as a major risk factor for introducing novel PRRSV strains into a herd, often leading to destabilization. Even for homegrown gilts, ensuring uniform immunity before they enter the breeding herd is critical to prevent the circulation of endemic strains to susceptible animals. Effective gilt immunization is therefore paramount for achieving herd stabilization and maintaining stability in PRRSV-positive farms [4]. The primary objectives are to ensure that gilts develop immunity to the resident PRRSV strains before entry into the breeding herd, thereby reducing the susceptible population pool and preventing virus transmission to pregnant sows [4].

Several strategies can be employed for herd stabilization, including natural exposure/cohabitating, positive serum inoculation, and vaccination with modified live virus (MLV) vaccines [4]. Although the MLV vaccination offers a safer, more controlled approach, it is not entirely risk-free. MLV strains have the potential to shed, which may result in a reversion to virulence or recombination with field strains [5,6,7]. Furthermore, these strains only provide limited cross-protection against genetically diverse heterologous field viruses [3,8,9]. While the primary criteria for selecting an MLV are its proven efficacy in preventing clinical disease and productive losses, the safety profile of the vaccine is also a critical consideration for long-term herd management. Provided that sufficient immunogenicity is achieved, an MLV that demonstrates a shorter duration of viremia and shedding may reduce the window of opportunity for recombination with field strains or potential reversion to virulence, thereby contributing to the overall biosecurity of the herd [8]. Therefore, the MLV with lower viremia and shorter shedding period should be chosen, leaving enough time for vaccinated gilts to “cool down” before they are introduced into sow herds to reduce the risk of recombination and reversion to virulence [8]. Besides, PRRSV infection itself, including vaccine virus replication, has been demonstrated to induce immunosuppression, which may in turn impair the pig’s overall health and response to other essential vaccines [10]. The complexity of PRRSV control is exacerbated by the frequent introduction of new viral strains into herds, often due to biosecurity challenges in high-density swine production regions like China and North America. This can lead to a dynamic and unpredictable landscape of the co-circulation of multiple, genetically distinct PRRSV strains. Once co-infection is established, the virus’s high rate of recombination can facilitate the generation of novel strains, which may become dominant in the population [11,12]. The predominant pattern of PRRSV in China is characterized by a recombination event between lineage 1 and lineage 8 [13]. The presence of this viral diversity has the potential to impede the efforts to acclimatize and may also compromise the efficacy of vaccines.

The complexity of PRRSV control is exacerbated by the virus’s dynamic epidemiology, characterized by high genetic diversity and the co-circulation of multiple strains, which can lead to recombination and the emergence of novel variants. To dissect such complex viral populations, advanced molecular tools are required. Next-generation sequencing (NGS) offers unprecedented depth for the analysis of viral evolutionary dynamics [14], thereby enabling the characterization of genetic diversity, the tracking of specific strain proportions, the identification of mutations, and the detection of recombination events within a host or farm, particularly in the case of complex mixed infection. The application of NGS dynamically throughout an intervention period can provide crucial insights into the impact of control measures on complex viral ecosystems [14]. The dynamic monitoring of multiple PRRSV strain proportions using NGS during an immunization program involving MLV and a pharmaceutical intervention has not been previously reported.

The complexity of managing PRRSV in field settings is often compounded by the presence of secondary bacterial pathogens, which necessitate therapeutic interventions. Tylvalosin, a third-generation macrolide antibiotic, is a relevant example. Tylvalosin is a derivative of Tylosin and modified by 3-acetyl-4-isovaleryl, with the chemical formula C_53_H_87_NO_19_ and a molecular weight of 1042.3. This compound has been observed to bind to the 50 s subunits of bacterial ribosomal, thereby inhibiting bacterial protein synthesis [3,15]. Tylvalosin is a pharmaceutical compound that has been reported effective to treat bacterial enzootic pneumonia in swine and infectious sinusitis in poultry [16,17]. However, it has also been reported to possess anti-inflammatory and immunomodulatory properties [18]. In addition to its primary antibacterial function, some in vitro studies have demonstrated that it can partially inhibit PRRSV proliferation and influence the course of viral infections with PRRS in vivo [19,20].

This study aimed to evaluate the effect of administration as an adjunct treatment during PRRSV MLV TJM immunization in gilts replacement on a farm endemically infected with both a classical (GD240101) and an HP-PRRSV-like (GD240102) PRRSV strain. In light of the routine immune procedure of TJM, an attenuated vaccine from parental HP-PRRSV strain TJ, and the high degree of nucleotide similarity between GD240102 and TJ strain, TJM was used as a vaccination strategy to achieve herd immunity, eliminate the wild-type strain, and maintain stability in PRRSV-positive farms. The objective of this study was to characterize the viral population dynamics in a PRRSV-endemic field setting where tylvalosin was administered during MLV immunization. Specifically, we aimed to use NGS to observe if and how this practice was associated with: (1) the duration of PRRSV viremia and shedding; (2) the intra-host dynamics and clearance rates of vaccine and field strains; (3) the uniformity of immune responses.

## 2. Materials and Methods

### 2.1. Farm Description and PRRSV Status

The study was conducted on a commercial 1500-sow, multi-phase, farrow-to-finish swine farm. The farm had a history of PRRSV outbreak, after which the virus became endemic, leading to persistent circulation and associated disease challenges, particularly in the nursery and finishing phases. This resulted in a high culling rate and poor performance among growing pigs. Prior molecular characterization based on ORF5 sequencing from samples collected across different production stages indicated the herd-level co-circulation of two distinct PRRSV-2 strains: GD240101 and GD240102.

### 2.2. Animals, Housing, and Experimental Design

One hundred clinically, homegrown healthy gilts, with approximately 120–130 days age, were utilized in the clinical trial. Sample size was determined based on previous studies and practical considerations for commercial farm trials. Given their origin from a PRRSV-endemic production flow, they were in a state of subclinical viral circulation, which was confirmed by baseline NGS analysis detecting the presence of endemic field strains (GD240101 and GD240102) in pooled serum samples at 0 dpv (see Results Section 3.1). Prior to the trial, these gilts had received a comprehensive farm vaccination protocol which involved multiple immunizations. This included vaccinations against PRRSV (TJM MLV administered at 15 days and 28 days of age), and CSFV (C strain MLV administered at 35 days, 60 days, and 105 days of age). Crucially, due to the endemic circulation of PRRSV on the farm, the gilts used in this study were considered immunologically non-naïve, having been exposed to both the farm’s vaccination program and circulating field strains prior to the trial. Gilts were randomly allocated into two treatment groups (*n* = 50/group): control and tylvalosin. The randomization sequence was generated by computer-based random number generator. The study was conducted in a partially blinded manner. On-farm personnel responsible for treatment administration (medicated feed) were aware of the group allocations. However, laboratory personnel performing sample analysis (RT-qPCR, ELISA, NGS) were blinded to the treatment groups, as samples were coded. The group identities were revealed only after all data analysis was complete. Each group had five pens, and the groups were housed in separate, environmentally controlled barns located over 300 m apart, with dedicated personnel and feeders to prevent cross-contamination. Standard management practices regarding stocking density, temperature, and ventilation were applied. All animal procedures were conducted in accordance with the ethical guidelines approved by the Laboratory Animal Ethical Committee of China Agricultural University (details are provided in the Institutional Review Board Statement). No animals suffered from serious illnesses or died during the experiment.

At day 0 post-vaccination (0 dpv) of the trial, all gilts in both groups received one intramuscular dose (as per label) of a commercial PRRSV MLV TJM vaccine. Starting from 2 dpv, gilts in the tylvalosin group received medicated feed containing tylvalosin tartrate premix (ECO-BIOK Animal Health, Wenzhou, Zhejiang, China) following instruction. The medication was administered cyclically: 15 consecutive days of medication followed by 15 days of non-medicated interval. The sequence was repeated for a total of three cycles, with medication period 2 dpv to 16 dpv, 32 dpv to 46 dpv, and 62 dpv to 76 dpv. The control group was administered the same basal diet without medication throughout the trial. No other macrolide antibiotics were utilized during the designated study period.

Saliva samples were collected daily from 1 dpv to 30 dpv using cotton ropes (one rope per pen, pooled by group), and then with weekly interval until the end of the observation period (12 weeks). Serum samples were collected from 10 randomly selected gilts per group on 0 dpv (pre-vaccination) and weekly thereafter for 12 weeks. Samples were subjected to a process of clarification or separation by centrifugation, after which they were stored at a temperature of −80 °C.

### 2.3. Nucleic Acid Testing and ELISA Antibody Testing

Viral RNA was extracted from 200 µL of serum or saliva using a nucleic acid extraction instrument. Subsequently, 5 μL of RNA was applied for the differential diagnosis of PRRSV by using a differential diagnostic kit designed to distinguish between classical (Lineage 5) and HP-PRRSV (Lineage 8) of the PRRSV-2 (Longkuo Suzhou Bioengineering, Suzhou, Jiangsu, China). The results were expressed as Ct values, of which under 36 were considered positive samples, and were shown as a positive rate.

Serum samples were tested for antibodies against PRRSV (IDEXX PRRS X3 Ab Test, IDEXX Laboratories, Westbrook, ME, USA) and Classical Swine Fever Virus (CSFV) (IDEXX CSFV Ab Test, IDEXX Laboratories, Westbrook, ME, USA). These two commercial ELISA kits were used following the manufacturer’s instructions. Antibodies of PRRSV and CSFV were continuously monitored weekly throughout the experiment, with these values expressed as either sample-to-positive (S/P) values (where a ratio of ≥ 0.4 was regarded as positive) or blocking rates (where an inhibition rate of ≥ 40% was regarded as positive). The coefficient of variation (CV) was calculated for PRRSV and CSFV antibody levels within each group at each time point (CV% = [Standard Deviation/Mean] × 100) to measure immune response uniformity [21].

### 2.4. Next-Generation Sequencing (NGS) and Bioinformatic Analysis

For each group and time point at which PRRSV RNA was detected by RT-qPCR, RNA extracts from the PRRSV-positive serum/silva samples were pooled by 50 μL/each, followed by NGS performed by Sangon Biotech (Shanghai, China) until week 9. In detail, total RNA was fragmented, followed by random primer reverse transcription and second-strand synthesis. Subsequently, 2 × 150 bp nucleotide paired-end sequencing was performed (Illumina, San Diego, CA, USA).

Raw sequencing reads were subjected to a quality filter using Trimmomatic, to eliminate Illumina adapters, with the default settings [22] being employed. Reads with under 36 base pairs (bp) in length were discarded, and the ends of read with low-quality bases (Q scores < 30) were trimmed. The alignment of the fastq files to the sequences of three co-circulating strains was conducted using the *ViReMa* Python script (Viral Recombination Mapper, version 0.29) [23]. Subsequently, the sequence alignment map (SAM) file was processed using samtools to ascertain the nucleotide depth at each position within a sorted binary alignment map (BAM) file, using the command line samtools depth -a -m 0 sample_virema.sort.bam > sample_virema.coverage.txt [22].

Phylogenetic analysis was conducted using the Maximum Likelihood (ML) method in MEGA12 (version 12.0.9), with the Kimura 2-parameter model selected according to the Bayesian Information Criterion (BIC). The bootstrap value was set to 500. The identification of potential recombination events was facilitated using the SimPlot (version 3.5.1) software. The GD240101, GD240102, and TJM (exhibiting 99.1% nucleotide similarity with the TJ strain, which was registered as EU860248.1 in Genbank) sequences were selected as the reference sequences for subsequent NGS analysis.

The relative abundance of each strain was estimated based on the proportion of uniquely mapped reads or average coverage depth. The average coverage depth (×) was calculated for each strain per sample. To ascertain the frequency of genomic junctions, a comparison was made between the nucleotides implicated in these junctions and the total mapped nucleotides. The number of nucleotides at these junction sites, as identified by ViReMa in the BED files, was aggregated for quantification. The total mapped nucleotide count was assessed using the sample_virema.coverage.txt file described above. Due to the variation in replication speed among different strains, junction patterns were plotted at approximately identical coverage levels. The coverage was determined by utilizing the seqkit software. The mutation rate of each virus was calculated by comparing the number of mutations to the aggregate number of nucleotides at each position in the genome. Mutations were selectively excluded from the calculation if their relative proportion was lower than 0.001. The total number of nucleotides was determined by aggregating the depth of nucleotides at each position throughout the genome, as indicated in the samtools-generated coverage files. Mutations were considered significant and recorded if their proportions were above 1%.

### 2.5. Statistical Analysis

The results of the antibody test were expressed as means ± standard deviations. All data were first tested for normal distribution using the Shapiro–Wilk test. For data following a normal distribution, comparisons between the two groups were performed using an unpaired Student’s *t*-test. For data that did not follow a normal distribution, the non-parametric Mann–Whitney U test was used. Comparisons involving multiple time points were analyzed using a two-way ANOVA with Sidak’s multiple comparisons test. The significance of the variability was determined using GraphPad Prism (version 10.4.0, GraphPad Software, San Diego, CA, USA) software. Asterisks indicate statistical significance; NS, no significance; *, *p * <  0.05; **, *p*  <  0.01; ***, *p*  <  0.001; ****, *p*  <  0.0001.

## 3. Results

### 3.1. Baseline Assessment Reveals Complex PRRSV Endemicity with Multiple Co-Circulating Strains

**Figure 1:**
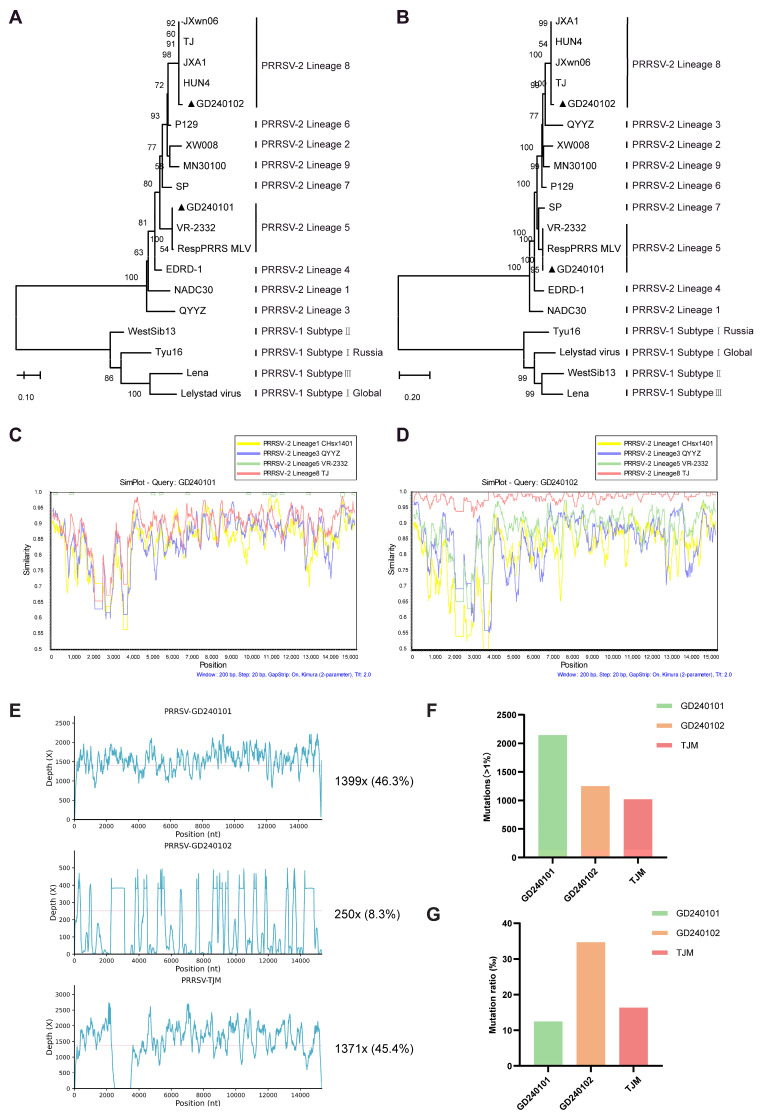
Baseline characterization of farm endemic PRRSV strains and pre-vaccination viral population. (**A**,**B**) Phylogenetic analysis based on ORF5 genes (**A**) and the complete genome sequences (**B**) of the farm isolates GD240101 and GD240102 (marked by ▲) alongside reference PRRSV strains. (**C**,**D**) Recombination analysis comparing the complete genomes of GD240101 (**C**) and GD240102 (**D**) against representative strains from different PRRSV-2 lineages using SimPlot. (**E**) Baseline average sequencing depth (×) detected by NGS in pooled serum and saliva samples from gilts before vaccination (0 dpv). (**F**) Baseline number of accumulated mutations (frequency > 1%) detected for each strain. (**G**) The baseline mutation ratio (‰) was calculated for each strain.

To identify the characteristics of co-circulated PRRSV strains on that farm before the trial, a phylogenetic analysis of ORF5 (Figure 1A) and the whole genome (Figure 1B) was conducted. The result demonstrated that GD240101 was clustered into PRRSV-2 lineage 5 (in the same branch as classical strain VR-2332 with 99.82% nucleotide similarity), and GD240102 was clustered to PRRSV-2 lineage8 (in the same branch as HP-PRRSV strain TJ with 98.29% nucleotide similarity), with no recombination signal within PRRSV-2 lineages (Figure 1C,D). Average sequencing depth by NGS analysis before the trial revealed the baseline proportion of GD240101 (46.3%), GD240102 (8.3%), and TJM (45.4%) in pigs (Figure 1E).

**Figure 2:**
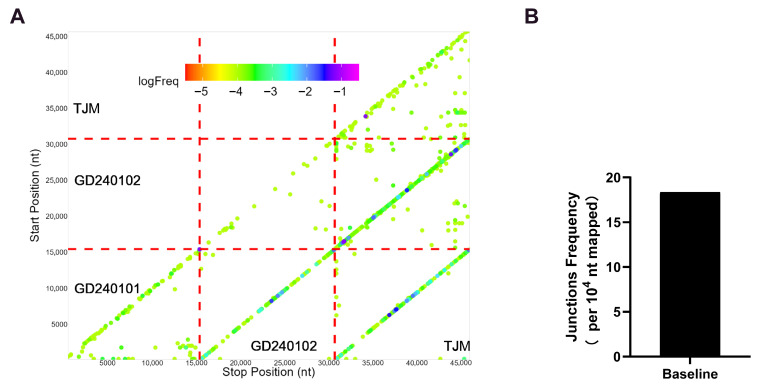
Recombination analysis of baseline infection. (**A**) The scatter plots are generated by junctions of three strains. Axes represent nucleotide start and stop positions. Red dashed lines delineate comparisons between different strains. Off-diagonal signals indicate potential recombination events (intra-strain within the three main diagonal blocks, inter-strain in the off-diagonal blocks). Each spot is colored according to its frequency in the population of all junctions, and the colors transition from a cool-toned to a warm-toned spectrum, corresponding to the frequency in all junctions from the highest to the lowest. (**B**) Calculated baseline recombination junction frequency (number of junction reads per 10^4^ mapped nucleotides) derived from the NGS data, quantifying the overall recombination activity before intervention.

Baseline mutations (Figure 1F) and mutation ratio (Figure 1G) demonstrate that, despite GD240101 exhibiting a higher number of mutation sites than GD240102, GD240102 manifests the highest mutation ratio. The accumulation of these mutations in these strains gives rise to high-abundance clusters and can result in recombination within or among strains due to the low RNA-dependent RNA polymerase (RdRp) fidelity of RNA viruses [24]. In addition to the presence of self-recombination (three regions of the nine-cell grid that belong to the same strain both horizontally and vertically), more recombination signals among strains have been observed in regions of the nine-cell grid that belong to different strains, both horizontally and vertically (Figure 2A). The baseline junction frequency was 18.34 per 10^4^ nt mapped (Figure 2B). The recombination baseline condition revealed a complex status of infection in pigs.

### 3.2. Clinical Sample Test by RT-qPCR Shows Tylvaloosin Promotes PRRSV Clearance in Pigs

**Figure 3:**
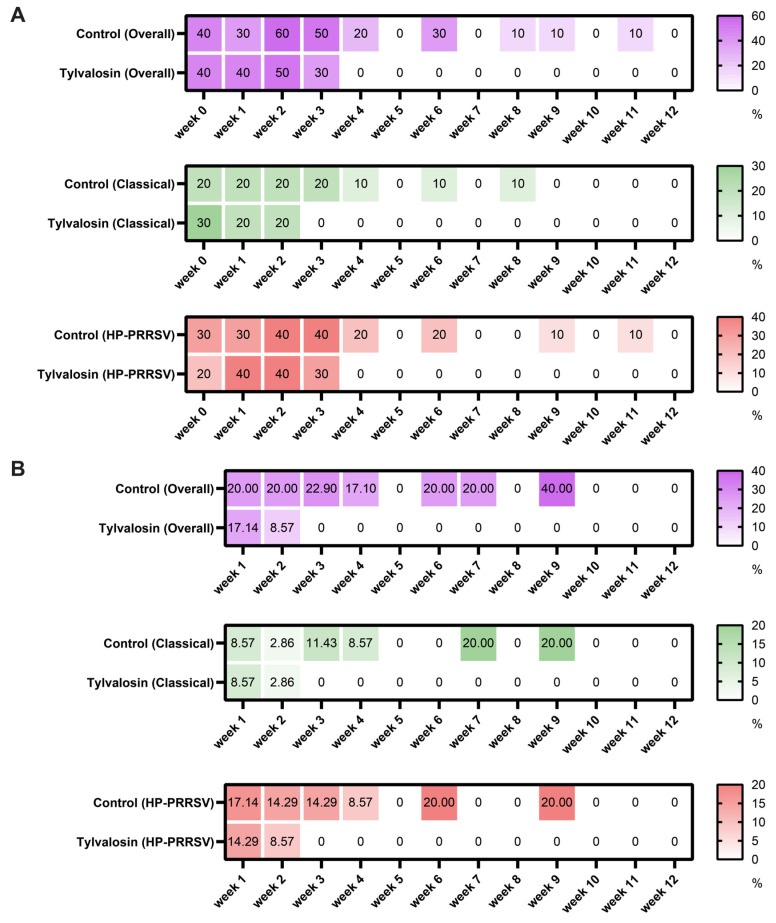
PRRSV detection rates in serum and saliva. Heatmaps showing the proportion (%) of PRRSV-positive samples detected by differential RT-qPCR in serum (**A**) and saliva (**B**) samples collected weekly from the control and tylvalosin groups over 12 weeks post-vaccination. Results are shown for overall PRRSV detection, detection of the classical strain type (corresponding to GD240101), and detection of the HP-PRRSV or HP-PRRSV-like strain type (corresponding to GD240102 and TJM). Color intensity represents the positive rate according to the scale bar (darker color indicates a higher positive rate). Weeks where no samples tested positive are shown in white.

Following vaccination, both groups demonstrated PRRSV viremia (Figure 3A, Tables S1 and S2) and salivary shedding (Figure 3B, Tables S3 and S4) in week 1. Moreover, the results of RT-qPCR, based on differential diagnostic kits showed that the positive rate of HP-PRRSV-like virus remained higher than that of classical PRRSV during the process of viral clearance. However, the proportion of PRRSV-positive samples decreased more rapidly in the tylvalosin group compared to the control group in both serum and saliva and was completely cleared by week 4 in serum as well as week 3 in saliva. The control groups exhibited negative outcomes at weeks 5, 7, and 10. Nonetheless, subsequent results did not indicate complete clearance of the virus. This may be due to false negative results from the randomly sampled 10 out of 50 pigs without positive samples. In more detail, the random sampling of ten pigs at weeks 8, 9, and 11 yielded a positive result in only one instance. (Note: Given the downward trend in viral titers, samples obtained after week 9 were not sequenced by NGS.) The above data suggests that the tylvalosin group achieved complete cessation of detectable viremia and shedding earlier than the control group.

### 3.3. NGS Analysis Demonstrates Accelerated Vaccine Strain Clearance and Altered Field Strain Dynamics Following Tylvalosin Usage

**Figure 4:**
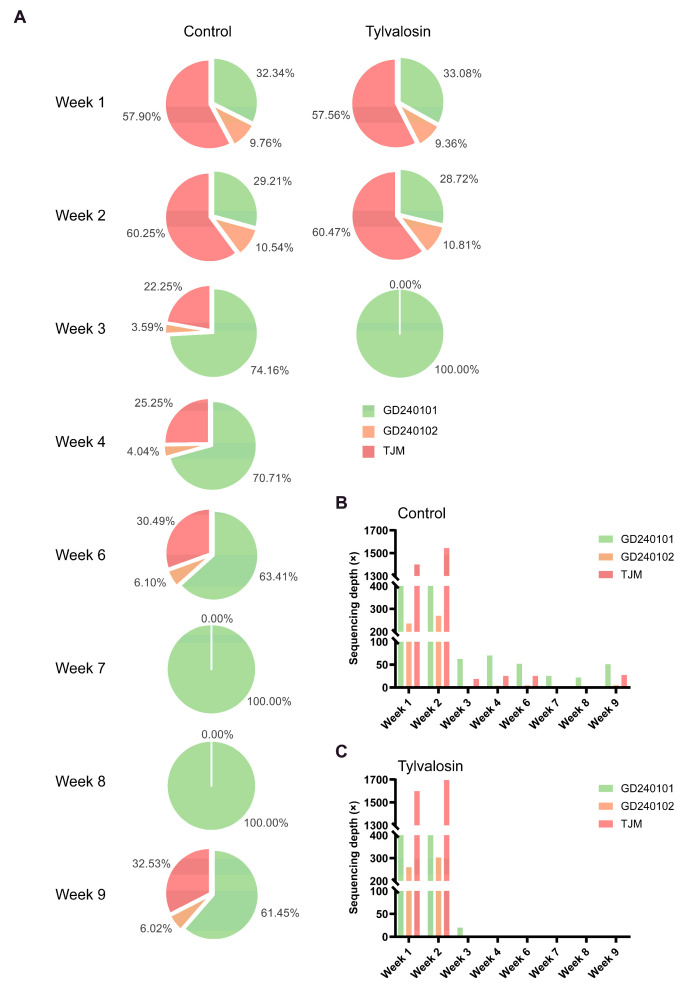
PRRSV strain dynamics monitored by NGS post-vaccination. (**A**) Relative abundance (%) of TJM, GD240101, and GD240102 were detected by NGS in pooled positive serum and saliva samples collected weekly from the control or tylvalosin groups. Strain proportions are represented by pie charts. (**B**,**C**) Average sequencing depth (×) for each detected strain (TJM, GD240101, GD240102) in positive pooled samples from the control group (**B**) or tylvalosin group (**C**) over the monitored weeks, reflecting the quantity of detectable viral RNA for each strain.

NGS analysis of pooled serum RNA revealed the dynamic changes in the relative abundance of the three target strains (GD240101, GD240102, TJM) (Figure 4A). In the initial phase (week 1 and week 2), the vaccine strain TJM rapidly established itself as the dominant strain detected in both the control and tylvalosin groups, accounting for approximately 57.90% and 57.50% of the viral population at week 1, respectively. The classical field strain GD240101 was the second most abundant, while the HP-PRRSV-like field strain GD240102 constituted a minor fraction during this period. A striking difference emerged by week 3. In the tylvalosin-usage group, the relative abundance of the TJM vaccine strain plummeted to undetectable levels (0.00%), indicating rapid clearance. Concurrently, the field strain GD240102 became the sole strain detected (100%) at this time point in the tylvalosin group, although the overall viral load was drastically reduced. The negative status was sustained in the tylvalosin group for all subsequent weeks under observation. In stark contrast, the TJM vaccine strain persisted in the control group, being detected at 4.00% in week 3, dropping further but still present at 5.70% in week 6, disappearing temporarily, and then reappearing at 5.85% in week 9. The dynamics of field strains were also found to differ in the control group. GD240102 became the predominant strain from week 3 (73.90%) through week 8 (100.00%), while GD240101 persisted alongside it until week 6 (30.70%) and reappeared with GD240102 and TJM in week 9 (32.21%). (Note: RT-qPCR results indicated samples were negative for PRRSV in week 5, hence no NGS data is presented for that time point.)

The average sequencing depth (raw data in Figure S1) of control (Figure 4B) and tylvalosin (Figure 4C) groups are indicative of detectable viral RNA. While both groups demonstrated a substantial decrease in overall viral RNA (lower depths for all strains) from week 3 onwards compared to weeks 1 and 2, the tylvalosin group exhibited a more profound and sustained reduction. Specifically, the depth for the TJM strain dropped to 20× by week 3 and remained at zero thereafter in the tylvalosin group (Figure 4C). In the control group, although depths decreased after week 2, low levels of TJM, GD240101, and particularly GD240102 RNA persisted intermittently through week 9 (Figure 4B), aligning with the relative abundance data.

**Figure 5:**
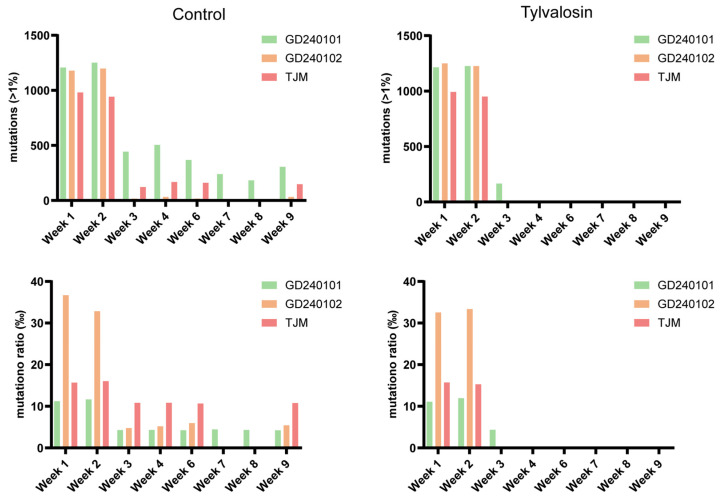
PRRSV mutation analysis by NGS post-vaccination. Including a total number of accumulated mutations (frequency > 1%) and mutation ratio (‰) for TJM, GD240101, and GD240102 strains over time in pooled samples from the control (left) and tylvalosin (right) groups. Bar colors indicate the respective strains.

The accumulation of mutations (frequency > 1%) and the genome-wide mutation ratio (‰) were analyzed over time for each detectable strain (Figure 5). In both groups, the number of detected mutations was highest during weeks 1 and 2, when viral loads (sequencing depths) were at their peak and decreased substantially. The GD240102 demonstrated a higher mutation ratio than the TJM and GD240101 in the initial weeks of the study in both groups. Nevertheless, no consistent or discernible disparities were identified in the number of accumulated mutations or the mutation ratios between the tylvalosin and control groups for any given strain during the periods in which they were reliably detected. This finding suggests that, within the specified timeframe and detection limits of this study, the tylvalosin usage did not appear to exert strong selective pressure, leading to substantial divergence in viral mutation patterns compared to the control group.

### 3.4. Tylvalosin Usage Correlates with Rapid Reduction in Detectable PRRSV Recombination Activity

**Figure 6:**
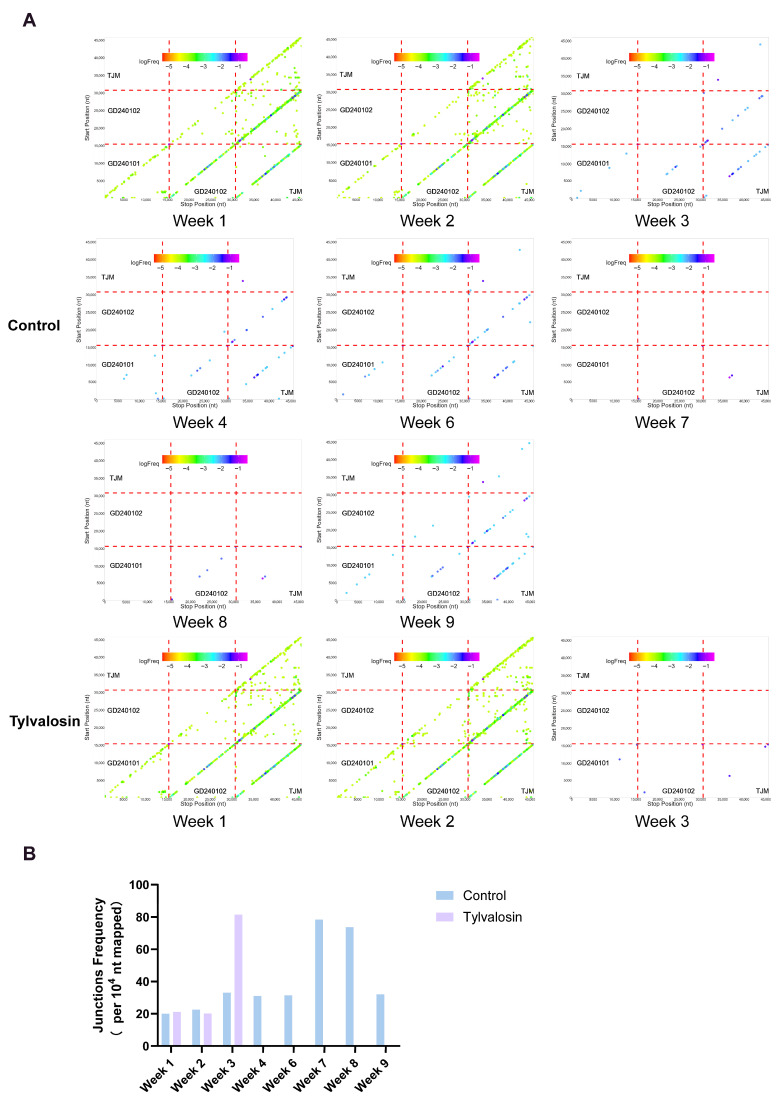
PRRSV Recombination Analysis by NGS Post-Vaccination. (**A**) Recombination scatter plots generated from NGS data of pooled samples. Off-diagonal signals indicate potential recombination events. Color intensity represents the logarithm of the recombination frequency (logFreq). (**B**) Calculated recombination junction frequency (number of junction reads per 10^4^ mapped nucleotides) over the monitored weeks for the control and tylvalosin groups.

To investigate the impact of tylvalosin on recombination during MLV immunization, we analyzed recombination patterns using NGS data. In the first two weeks following MLV vaccination, which corresponded to the peak viremia and highest viral loads detected, recombination activity remained prominent in both experimental groups. The dot plots for week 1 and week 2 (Figure 6A) exhibited a resemblance to the baseline complexity before vaccination, displaying numerous off-diagonal recombination signals across both intra- and inter-strain comparison regions. This finding suggests that the introduction and subsequent rapid replication of the TJM vaccine strain, in conjunction with the existing field strains provided an ample opportunity for genetic exchange. The calculated junction frequencies, raised from 18.34 junctions/104 nt, reflected this activity and were comparable between 20.04 junctions/104 nt (control) versus 21.15 junctions/104 nt (tylvalosin) at week 1, and 22.51 junctions/104 nt (control) versus 20.12 junctions/104 nt (tylvalosin) at week 2 (Figure 6B). This initial similarity highlights that both groups initiated with comparable levels of active viral replication and associated recombination following vaccination. However, a marked divergence emerged from week 3 onwards, coinciding with the divergent viral clearance patterns observed between the groups. In the control group, evidence of ongoing recombination persisted throughout the 9-week observation period with dot plots consistently showing numerous off-diagonal signals.

The calculated junction frequency remained elevated, ranging from 31.04 to 33.02 junctions/10^4^ nt during weeks 3, 4, 6, and 9, and notably peaking at 78.40 (week 7) and 73.69 (week 8) junctions/10^4^ nt, reflecting the continued co-circulation and dynamic interplay of the TJM, GD240101, and dominant GD240102 strains in these animals. However, as illustrated in Figure 6A, it is intuitive to see those parental strains (i.e., strains lacking inter-strain recombination) declined particularly from week 3, suggesting that parental strains were unable to evade immune pressure. Hence, very few dots were plotted on the diagonal line. Analogously, the tylvalosin-usage group exhibited a dramatic reduction in detectable recombination activity starting at week 3. The corresponding dot plots appeared markedly sparse, correlating with the rapid clearance of the TJM and GD240102 strains and the drastically diminished overall viral load achieved in this group. While an anomalously high calculated junction frequency (81.45 junctions/104 nt) was recorded for the tylvalosin group at week 3, this occurred simultaneously with a sharp drop in sequencing depth. This further elucidates that among the residual GD240101 strains identified at this time (Figure 4), the strains that underwent recombination with GD240101 and the other two strains accounted for a high proportion, and these recombinants were cleared in the following week. Therefore, the overall analysis indicates that while recombination was active initially in both groups, administration of tylvalosin was associated with rapid cessation of detectable recombination activity, paralleling the accelerated viral clearance. In contrast, the control group maintained a dynamic recombination profile associated with persistent viral co-circulation.

### 3.5. Tylvalosin Usage Enhances the Uniformity of PRRSV and CSFV Antibody Responses

**Figure 7:**
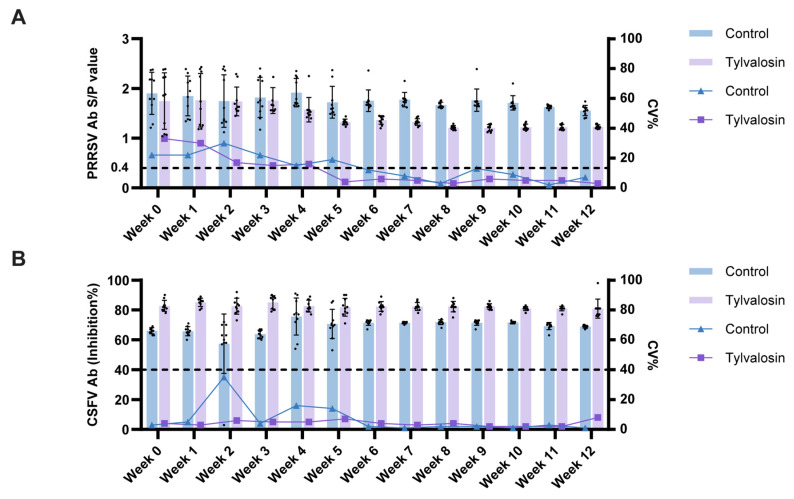
Humoral immune responses to PRRSV and CSFV. Kinetics and uniformity of serum antibody responses over 12 weeks post-vaccination for the control and tylvalosin groups. (**A**) PRRSV antibody S/B value (bars and points, left y-axis) and CV% (line, right y-axis). Dashed lines indicate positive cutoff values of 0.4. (**B**) CSFV antibody inhibition rate (bars and points, left y-axis) and CV% (line, right y-axis). Dashed lines indicate positive cutoff values of 40%.

To assess the impact of tylvalosin usage during PRRSV MLV immunization on the humoral immune response to PRRSV and other critical swine pathogens, serum antibody kinetics and endpoint levels were evaluated using ELISA. The uniformity of the response within each group was assessed by calculating the CV%. Both groups seroconverted to PRRSV positive before vaccination (Figure 7A, Table S5). The mean levels increased comparably in both the control and tylvalosin groups, plateauing around weeks 4–5. After this, the mean S/P values of the tylvalosin group were found to be marginally lower than those of the control group. While both groups demonstrated a decline in CV% following seroconversion, the tylvalosin group achieved lower variability at an earlier stage and maintained this lower CV% (<10%) from week 5 onwards, in comparison to the control group, where CV% remained higher and more variable. This indicated that tylvalosin usage led to a more uniform systemic antibody response to PRRSV within the group, likely reflecting the cumulative immunity from both vaccination and field virus exposure. The mean CSFV antibody levels were established at week 0 in both groups and remained relatively stable throughout the study (Figure 7B, Table S5). The tylvalosin group demonstrated marginally elevated mean inhibition rates in comparison to the control group. Regarding uniformity, the tylvalosin group displayed consistently lower variability (CV% < 10%) compared to the control group, which showed higher initial CV%. This suggests that tylvalosin usage was associated with a more uniform CSFV antibody profile.

## 4. Discussion

This study investigated the viral and immunological factors in a PRRSV-endemic farm setting where tylvalosin, a macrolide antibiotic used to manage secondary bacterial pathogens, was administered during PRRSV MLV (TJM strain) immunization program. In this context, our primary observation was that this intervention was associated with an accelerated clearance of both the vaccine strain and a co-circulating highly pathogenic field strain (GD240102). Furthermore, this intervention was correlated with a more uniform systemic antibody response to PRRSV and CSFV. The application of dynamic NGS monitoring has provided novel insights into the complex interplay between the vaccine virus, field viruses, and the intervention strategy.

The most striking virological finding was the rapid clearance of the TJM vaccine strain observed via NGS in the tylvalosin group, becoming undetectable by week 3 post-vaccination, compared to its persistence until week 9 in controls. This accelerated clearance was corroborated by the earlier cessation of viremia and shedding, as detected by RT-qPCR. Although tylvalosin does not target PRRSV directly, it can achieve an antiviral effect through its long-term action on the cells during the PRRSV proliferation [25], due to its anti-oxidative and anti-inflammatory activity [18] and its ability to suppress the PRRSV-induced NF-κB activation and cytokine expression [26]. Tylvalosin might exert immunomodulatory effects to improve the host’s immunological condition, attenuated the inflammatory response, increased monocyte counts, elevated serum IFN-γ concentrations, and attenuated the reduction in CD8+ T cells, thereby allowing for a more efficient innate and adaptive immune response, which in turn led to faster viral clearance [27]. From a biosafety perspective, reducing the duration of vaccine virus shedding is often considered a strategy to minimize the window of opportunity for recombination with field strains, though this must be carefully balanced against the need to ensure sufficient duration of antigen presence for robust immunization. There is a fundamental trade-off in MLV immunology between safety and immunogenicity. Sufficient viral replication and persistence are required to adequately stimulate the host immune system and induce a robust, long-lasting protective immunity. An overly rapid clearance of the vaccine antigen could potentially hamper the development of this immune memory. This study focused on the virological dynamics and did not assess the long-term protective immunity of the gilts. Therefore, while our data shows a benefit in terms of viral clearance duration, we cannot conclude whether this came at the cost of reduced immune potency. Determining the optimal balance between minimizing shedding risks and maximizing protective immunity is a critical challenge in swine health management and warrants further investigation.

In addition, it is imperative to frame these findings within the principles of responsible antimicrobial stewardship. Tylvalosin is a medically important antimicrobial, and its use should be strictly reserved for the therapeutic treatment of diagnosed bacterial diseases under veterinary supervision. The prophylactic use of antimicrobials for viral disease control or performance enhancement is contrary to global health guidelines (e.g., WHO, FAO) and poses a significant risk of promoting antimicrobial resistance. The observations in this study should therefore not be interpreted as a recommendation for such use. Instead, they highlight a complex drug–host–virus interaction that warrants further mechanistic investigation.

The NGS analysis of mutation and recombination dynamics did not reveal major differences between the groups within the 12-week timeframe. This finding may imply that the administration of the short-term, cyclic tylvalosin usage did not exert significant selective pressure, resulting in the evolution of the virus as evidenced by pooled samples, or that such events were infrequent occurrences. Longer-term monitoring or individual animal sequencing might be required to detect subtle evolutionary effects. However, the observation of a similar dynamic recombination profile, characterized by a high junction frequency, in both groups before the clearance of the virus, suggests that many recombinants or defective virus genomes (DVGs) may be the viruses’ “evolutionary dead-ends” before being eliminated under the pressure of immune response. It is crucial that our NGS-based method detects recombined RNA segments and does not provide direct evidence for the production of viable, infectious recombinant virions. The cessation of detectable recombination in the tylvalosin group should be viewed as an indirect consequence of the usage. While our calculation of a normalized junction frequency was designed to account for variations in sequencing depth, the near-complete elimination of the viral template is the most plausible reason for the diminished opportunity for recombination

An immunological finding in this study was the improved uniformity of systemic antibody responses to PRRSV and CSFV in the tylvalosin group, evidenced by lower CVs for PRRSV and CSFV antibodies throughout much of the study period. The CV reflects the variability of response within a group; a lower CV suggests that gilts in the tylvalosin group responded more consistently to overall immunity to PRRSV, which is the combined result of exposure to both the vaccine and the co-circulating field strains. PRRSV is notable for its immunomodulatory and frequent immunosuppressive effects, which can result in variable responses to PRRSV itself and concurrently administered vaccines [28]. By facilitating faster PRRSV clearance, tylvalosin may have mitigated these negative immunological effects, relieved its inhibition to CSFV replication via GSDMD-mediated pyroptosis, hence improved CSFV antibody levels [29].

This study uniquely employed dynamic NGS to dissect the complex interactions between an MLV vaccine, two distinct field strains, and a pharmaceutical intervention during gilt immunization. Previous studies have evaluated the efficacy of MLV or tylvalosin for PRRS-associated respiratory disease, but none have integrated these elements with deep sequencing to monitor strain-specific dynamics. The present findings build upon the findings of previous studies by observing that a tylvalosin adjunct therapy can influence vaccine and field virus kinetics and improve immune response consistency.

Limitations of this study must be carefully considered when interpreting the results. The most fundamental limitations relate to the study design and the context of the animal population. The experiment was conducted with immunologically non-naïve, pre-exposed gilts, meaning our findings cannot be extrapolated to naïve animals where the host–virus interaction might differ. Furthermore, the study lacks a control group treated with a different antimicrobial, which prevents us from definitively separating any specific anti-PRRSV effects of tylvalosin from the general, confounding benefits of controlling secondary bacterial infections. These design parameters mean our study should be viewed as a characterization of an intervention under specific, complex field conditions rather than a controlled efficacy trial.

Several methodological aspects also warrant consideration. The weekly sampling of only 10 out of 50 gilts may have introduced selection bias and potential false negatives. Similarly, the potential for minor environmental contamination of oral fluid samples cannot be entirely ruled out. The use of pooled samples for NGS, while providing a valuable population-level perspective, obscures individual animal variation and precludes statistical comparisons of viral recombination dynamic metrics. Finally, the scope of this study was focused on virological and immunological dynamics; the precise mechanisms of tylvalosin’s action and, critically, its impact on subsequent reproductive performance were not assessed. While the observed improvements in viral clearance and immune uniformity are biologically expected to lead to better production outcomes, this correlation was not empirically tested in this trial. Future studies are therefore warranted to connect these virological and immunological phenomenon to tangible improvements in on-farm productivity.

## 5. Conclusions

In conclusion, this field study observed that in gilts receiving tylvalosin for the management of bacterial pathogens during PRRSV MLV (TJM strain) immunization in pre-exposed replacement gilts during a PRRSV MLV immunization program, there was an associated accelerated clearance of both vaccine and co-circulating field strains (GD240101 and GD240102) and a more uniform systemic antibody responses against PRRSV and CSFV. These findings, characterized by NGS, describe a complex drug–host–virus interaction in a field setting. Crucially, while this study reveals a complex biological interaction, these findings must not be interpreted as an endorsement for the prophylactic use of antimicrobials. In line with antimicrobial stewardship, tylvalosin’s use should be strictly therapeutic for bacterial diseases to mitigate resistance risks. The interpretation of these findings should also consider the study’s key limitations, namely the use of pre-exposed animals and the lack of an alternative antimicrobial control group.

## Data Availability

The PRRSV strains TJ mentioned in this study are available in GenBank with the accession numbers EU860248.1.

## Supplementary Materials

The following supporting information can be downloaded at: https://www.mdpi.com/article/10.3390/vaccines13101007/s1, Figure S1: Average sequencing depth (×) detected by NGS in pooled serum and saliva samples from gilts post-vaccination; Table S1: RT-qPCR serum test; Table S2: RT-qPCR serum test positive rates; Table S3: RT-qPCR saliva test; Table S4: RT-qPCR saliva test positive rates; Table S5: Dynamic monitoring of antibodies.
